# Prognostic Value of Selective Nerve Root Blocks Prior to Pulsed Radiofrequency in the Treatment of Patients With Chronic Radicular Pain: A Systematic Review

**DOI:** 10.1111/papr.70132

**Published:** 2026-03-06

**Authors:** Marius R. van Ooijen, Sezai Özkan, Koen van Boxem, Kris C. P. Vissers, Sandra A. S. van den Heuvel

**Affiliations:** ^1^ Department of Anesthesiology, Pain and Palliative Medicine Radboudumc Nijmegen the Netherlands; ^2^ Department of Anesthesiology Intensive Care Medicine, Emergency Medicine and Multidisciplinary Pain Center, Ziekenhuis Oost‐Limburg Genk/Lanaken Belgium

## Abstract

**Background/Importance:**

Selective nerve root blocks (SNRBs) are frequently used in clinical algorithms for managing chronic radicular pain. However, their prognostic value in identifying patients likely to benefit from pulsed radiofrequency (PRF) treatment remains uncertain.

**Objective:**

This systematic review evaluates whether a positive response to an SNRB predicts improved clinical outcomes following PRF in patients with chronic radicular pain.

**Evidence Review:**

A systematic search was conducted in PubMed, Embase, and Cochrane databases, along with reference lists of relevant articles. Eligible studies included patients with chronic radicular pain and assessed the prognostic role of SNRBs administered prior to PRF. Risk of bias was assessed using the ROBINS‐I V2 tool.

**Results:**

Only one prospective observational study met inclusion criteria. In patients with chronic lumbosacral radicular pain, a positive SNRB response was associated with a higher likelihood of treatment success at 6‐week follow‐up (odds ratio: 3.26; 95% CI: 0.97–11.00; *p* = 0.06). Multivariate analysis identified limited baseline disability, age > 55 years, and a positive SNRB response as predictors of success at 6 months, with an area under the receiver operating characteristic curve of 0.73.

**Conclusions:**

This review identified a lack of published studies—aside from one prospective observational study—examining the prognostic value of SNRBs in the context of PRF for chronic radicular pain. The findings underscore not only a lack of high‐quality evidence but a broader gap in the literature. Further robust research is warranted to clarify the clinical utility of SNRBs in guiding PRF treatment decisions.

## 1 Introduction

Radicular pain—with or without accompanying radiculopathy—is a common clinical problem and is characterized by radiating pain in one or more dermatomes, with or without other radicular symptoms or decreased sensory and/or motor function. Radicular pain can arise at any level but is most commonly observed in the lumbosacral region, with a lifetime prevalence up to 43%, and far less in the thoracic region [1, 2, 3]. In most patients, pain resolves with no or conservative therapy within 6 months [4, 5]. For those patients with persistent symptoms, current international guidelines recommend performing pulsed radiofrequency (PRF) of the dorsal root ganglion preceded by a prognostic selective nerve root block (SNRB) in patients with persistent symptoms (> 12 weeks) [4, 5, 6]. The goal of these SNRBs is to guide patient selection by identifying suitable patients for successful treatment with PRF.

Prognostic SNRBs are performed under local anesthesia and fluoroscopic guidance by placing a needle in the intervertebral foramen at the suspected causal symptomatic level. A small volume of contrast agent is injected to confirm correct needle positioning adjacent to the nerve root and to rule out intravascular spread. Hereafter, 0.5–0.8 mL of a short‐acting local anesthetic is injected. The prognostic SNRB can be considered positive if pain measured by the numeric rating scale (NRS) decreases by 2 points or more or by more than 50% within 30 min after the intervention [5]. Patients with a positive response are scheduled for PRF treatment of the accordant dorsal root ganglion. A positive SNRB may contribute to improved clinical outcomes following PRF, and a negative response may help avoid ineffective or unnecessary interventions.

Although SNRBs are commonly included in clinical treatment algorithms worldwide, their prognostic value in selecting patients for PRF remains uncertain. The specificity of SNRBs is limited [7, 8], and the clinical effect of a SNRB can be difficult to interpret in patients with chronic symptoms [7]. Accuracy of prognostic SNRB is dependent on technical factors such as needle tip positioning and volume of local anesthetic used [9, 10]. Unintended spread to adjacent structures—including other nerves, facet joints, ligaments, muscles or intervertebral discs—may reduce specificity. In addition, beyond these clinical considerations on validity, the routine use of SNRBs may warrant critical evaluation from a health‐economic perspective. Healthcare systems are increasingly challenged by expanding costs, shortage of medical professionals and an aging population with increasing comorbidity burden [11]. In this health‐economic context, the added value of routinely performing prognostic SNRBs in advance to treatment with PRF of the dorsal root ganglion in patients with chronic radicular pain needs to be justified by evidence.

In conclusion, this systematic review aims to assess the evidence whether a positive response to a SNRB predicts improved clinical outcome after PRF.

## 2 Methods

This systematic review was performed following the PRISMA guidelines [12]. The review methodology including eligibility criteria, data items, and planned analysis strategy was pre‐specified and registered in PROSPERO (registration number CRD42024490918, 18 January 2024) [13].

The following PICO was used: (P) Adults with chronic radicular pain undergoing PRF of the dorsal root ganglion; (I) SNRB preceding treatment with PRF; (C) No SNRB preceding treatment with PRF; (O) Clinical effectiveness of PRF on pain, functioning, and quality of life.

### 2.1 Eligibility Criteria

Studies were included if they met all of the following criteria: (1) the study was an original full paper which presented unique data; (2) the study was performed in adult patients with suspected radicular pain on cervical, thoracic, or lumbosacral level (supported by history and/or physical examination and/or imaging); (3) the study discussed the value of a prognostic SNRB preceding a PRF adjacent to the dorsal root ganglion.

### 2.2 Information Sources

PubMed, Embase (including trial registries and conference abstracts), and Cochrane were searched to identify all studies evaluating the relation of a positive SNRB on the effectiveness of PRF in patients with chronic radicular pain. Reference lists of included articles and relevant reviews were screened for additional eligible studies.

### 2.3 Search Strategy

The full search strategy (see Appendix S1) was based on the (combination of) search components “dorsal root ganglion”, “pulsed radiofrequency treatment”, “prognostic”, and “radicular pain”. The search strategy was constructed in cooperation with a librarian of Radboud University. Reference lists of included studies and relevant reviews identified by our search were checked for additional eligible references. The search was performed in February 2024 and updated in October 2024, May 2025 and October 2025.

### 2.4 Selection Process

Search results from all databases were imported in reference manager software, RAYYAN [14] and duplicates removed. Title and abstract were initially screened and obvious irrelevant papers were excluded. Full‐text copies of all publications eligible for inclusion were subsequently assessed. Titles, abstracts and full texts were assessed by three of the authors independently (M.O. and S.Ö. or S.H.). Discrepancies were resolved through discussion until consensus was reached. A standardized inclusion form was used during the screening process.

### 2.5 Data Items

Bibliographic details such as author, journal, and year of publication, as well as data on study characteristics were extracted: (1) demographic data (e.g., age, gender, duration of complaints); (2) clinical data (e.g., selection criteria for interventional therapy, interventional procedures); and (3) outcome measures: pain intensity (e.g., NRS, visual analogue scale or others) was considered the primary outcome, whereas analgesic medication use (e.g., opioid in morphine milligrams equivalent (MME), neuropathic medication), patient functioning (e.g., ODI), return to work and quality of life (e.g., RAND‐36) were secondary outcomes.

### 2.6 Study Risk of Bias Assessment

Risk of bias of selected studies was assessed by two authors independently (M.O. and S.H.) by using the ROBINS‐I V2 tool (launch date 22 November 2024) [15]. Disagreements were resolved through consensus. The Robvis tool was used for visualizing the outcome of this assessment [16].

## 3 Results

### 3.1 Study Selection

A total of 252 records were identified through database searches (PubMed, *n* = 55; Embase, *n* = 166; Cochrane, *n* = 31). After removing 54 duplicates, 198 records remained for title and abstract screening. Of these, five studies were assessed for full‐text review, of which one study turned out to be a conference abstract only. Three out of four of the remaining studies were excluded for various reasons [17, 18, 19] described in Table 1.

**TABLE 1:** Reason for exclusion of excluded studies.

Study	Reason for exclusion
Wang, 2017	Compared effectiveness of three therapy strategies (SNRB, SNRB + PRF and PRF alone) in patients with chronic cervical radicular pain. SNRB included steroid injection and was not performed prior to the PRF, and thus not as a selection tool for optimizing outcome of PRF.
Lee, 2018	Assessed patients with primarily chronic low back pain, not chronic radicular pain.
De, 2020	Assessed therapeutic effectiveness of PRF versus local anesthetic rather than prognostic value of preceding SNRB.

**FIGURE 1:**
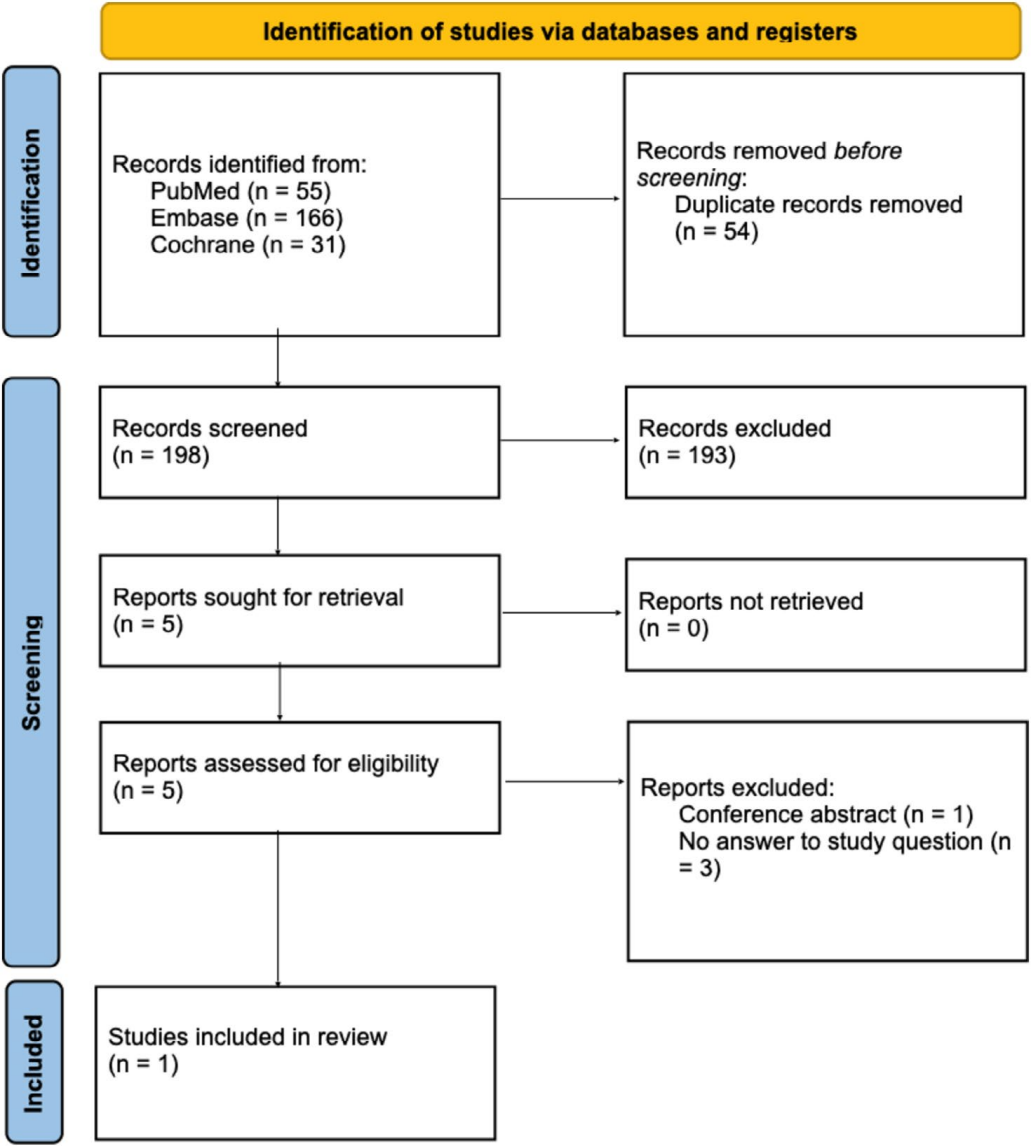
PRISMA chart of study selection.

Ultimately, we were able to include only one study that answered our PICO‐question. The study selection process is summarized in the PRISMA flow diagram (Figure 1).

### 3.2 Characteristics of the Included Study

The included study was a prospective observational study that aimed to identify predictive factors for successful treatment with PRF in patients with lumbosacral radicular pain [20].

Inclusion criteria were lumbosacral radicular pain (L5 or S1 level) for more than 3 months, despite conservative treatment. Pain intensity had to be a NRS of more than 4. Medical history had to be in accordance with imaging (CT or MRI).

All patients received a prognostic SNRB with a local anesthetic, followed by PRF of the same level (L5 or S1) based on clinical presentation and imaging. No control group without preceding SNRB was included. A positive prognostic SNRB was defined as an NRS reduction of 2 or more points or at least 50% reduction within 30 min after the block.

Baseline measurements included pain scores (NRS), analgesic use (opioid use, Medication Quantification Scale (MQSIII)), functional status (ODI), quality of life (RAND‐36), and neuropathic character of pain (Douleur Neuropathique 4, LANSS pain scale). NRS and Global Perceived Effect (GPE) were assessed 6 weeks, 3 months, and 6 months after treatment with PRF.

A positive outcome of PRF was defined as at least 50% pain relief on the GPE or more than 2 point reduction on the NRS of 2. Uni‐ and multivariate analysis was performed on all outcome parameters.

### 3.3 Risk of Bias

**FIGURE 2:**
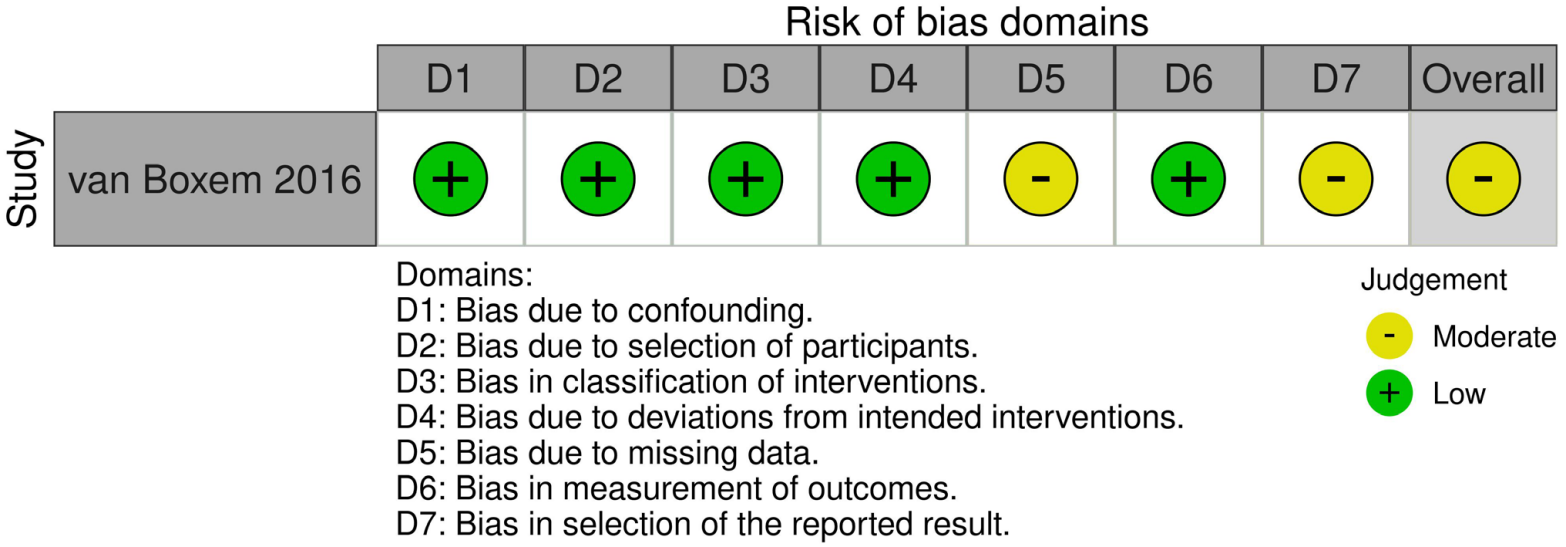
Risk of bias domains.

**FIGURE 3:**
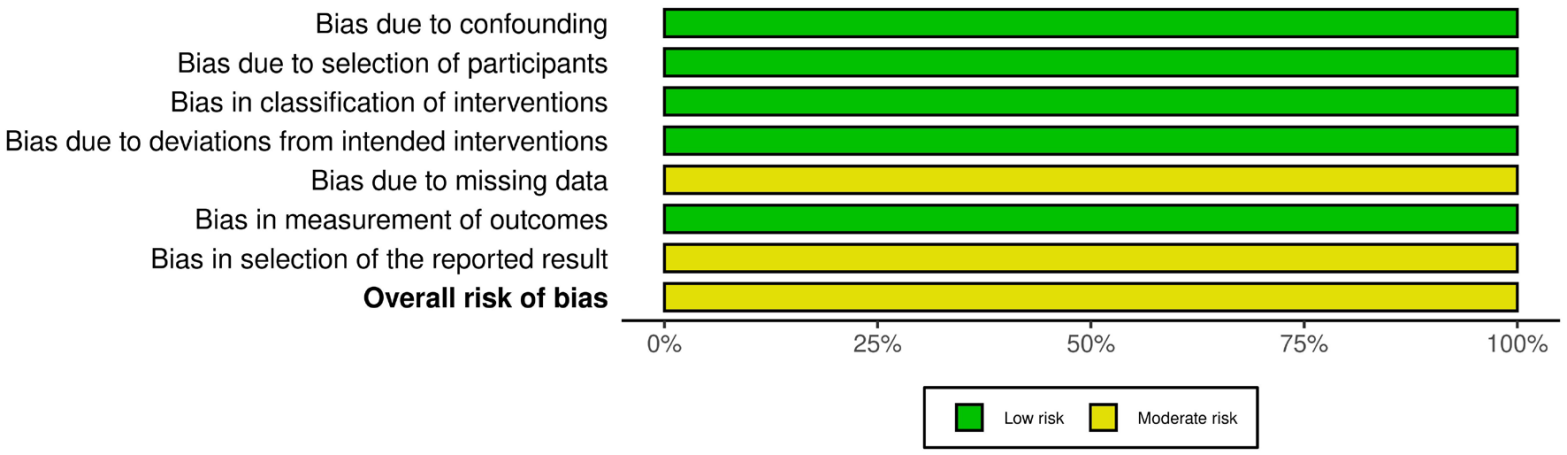
Risk of bias domains presented as percentages across the included study.

Assessment of risk of bias of the included study is summarized in Figures 2 and 3. Overall risk of bias was judged as “moderate.”

### 3.4 Results of Included Study

A total of 65 patients were included (27.7% male, 72.3% female; age ≥ 55 years old in 43.1%). Duration of symptoms ranged from 3 months to more than 1 year.

In univariate analysis, a positive prognostic SNRB was associated with a higher likelihood of treatment success with PRF at 6 weeks follow up, with an odds ratio of 3.26 (95% CI 0.97–11.00; *p* = 0.06).

Multivariate analysis identified a combination of limited baseline disability (ODI), age > 55 years, and a positive prognostic SNRB as predictive for treatment success with PRF at 6 months, with an area under the receiver operating characteristic curve of 0.73.

Some patients with a negative prognostic SNRB still experienced clinically relevant improvement after PRF, depending on the outcome measure used. Based on the NRS, there were three false‐negative results, while the GPE indicated a total of 14 false negatives. When combining both NRS and GPE, five patients were identified as having a false‐negative response.

## 4 Discussion

This systematic review investigated the effect of prognostic SNRBs on the effectiveness of PRF on pain, functioning, and quality of life in patients with chronic radicular pain. We found only one study meeting the inclusion criteria of this systematic review. In this study, a positive SNRB was associated with reduced pain at 6 weeks. When combined with limited disability and age > 55 years, a positive prognostic SNRB showed fair predictive value for an analgesic effect at 6 months follow‐up. Notably, some patients with a negative prognostic SNRB also experienced favorable outcomes after PRF, indicating potential false‐negative responses to SNRB.

Other studies, in which prognostic SNRBs as a predictive tool for therapy outcome were used, focused on the value of these SNRB for predicting successful outcome after decompression surgery in patients with radicular pain. These studies were analyzed in a systematic review [8]. A pooled analysis with post‐surgery outcomes as the reference standard, demonstrated that specificity of SNRBs was 22% (7.45%–49.9%), and sensitivity was 90.9% (83.1%–95.3%). All included studies had a high risk of bias. Three out of six studies were retrospective in nature, of which in two studies only patients with discordant imaging and clinical findings were included. In 5 out of 6 six studies 1 mL or more (up to 3 mL) local anesthetic was injected, which is not in agreement with international guidelines [5]. Level of injection was not reported in two studies and varied between L1 to S1 in the other studies. Therefore, it is unclear whether SNRBs are of value in improving patient outcomes after lumbar decompression surgery.

### 4.1 Limitations of Evidence in the Review

The primary aim of the observational prospective study included in this systematic review was to evaluate the effectiveness of PRF in patients with chronic radicular pain and to identify predictive factors for a successful outcome of PRF using a univariate and multivariate regression model. The prognostic value of a SNRB on outcome after PRF was not the primary focus of this study. All patients received a prognostic SNRB, were not randomized, and as such no comparison could be made with a group receiving PRF without such a selection step. The independent prognostic value of SNRB therefore remains unclear.

In this study, selection criteria were strict to create a homogeneous study group (only patients with monosegmental radiating pain in L5 or S1). Patients with painful radiation in more than one dermatome were excluded from the study. However, it is known that dermatomal mapping is dependent on the technique used, that there is a large overlap in dermatomes L5 and S1, and that the indicated dermatome often does not respond to the anatomically affected nerve root [21]. Consequently, there was an important risk of exclusion from this study resulting in the treatment of the non‐affected anatomical level with both the SNRB and the PRF.

This systematic review found no studies in which SNRB was evaluated in treatment with PRF of patients with chronic radicular pain on a cervical or thoracic level. It might be possible that SNRBs are of different value in patients with chronic cervical, thoracic, and lumbosacral radicular pain.

### 4.2 Limitations in the Review Process and Potential Impact of Each Limitation

The most obvious limitation of this review is that only one study could be included. Based on this study, it is hard to make final conclusions about the value of SNRBs in selecting patients for successful treatment with PRF. However, the fact that only one study was included does not downgrade the methodological quality of this systematic review. Also, underlining that only one study was included, the systematic search strategy and adherence to PRISMA methodology confirmed that there is limited published data to support evidence on this topic. This underscores a substantial gap in the scientific literature regarding the prognostic utility of SNRBs in the context of PRF. A final limitation is that an author of the included study is also co‐author of this systematic review. He was intentionally not involved in screening of identified studies and did not play a role in the assessment of the risk of bias for that study. We therefore negated any potential impact on the outcomes of this review.

### 4.3 Implications for Practice and Policy

Current guidelines recommend performing PRF of the dorsal root ganglion after a positive prognostic SNRB as a next step after conservative treatments in patients with chronic radicular pain [4, 5, 6]. However, this systematic review identified only one study evaluating the prognostic value of SNRBs prior to PRF and this study lacked a comparator group without SNRB. Furthermore, it is unclear if identifying a positive SNRB in a strictly homogenous study group of patients with chronic radicular pain in the lumbosacral region justifies extrapolation to patients with chronic radicular pain on thoracic or cervical level and to the heterogenous group of patients found in daily practice.

Although a positive prognostic SNRB was associated with improved short‐term outcomes in the included study, several patients with a negative prognostic SNRB still experienced clinical benefit from PRF. In general, technical variability, placebo effects, or patient factors like pain chronicity might influence the outcome of treatment with PRF after false negative SNRBs. In the included study, the authors found that false negative SNRBs depended on clinical outcome parameters used (GPE, NRS, or both). This urges the scientific society to start high quality prospective studies to demonstrate the validity and the discriminative value of prognostic SNRBs. Hence, also validating the health‐economic cost of these procedures to prove the best possible clinical outcome for the most appropriate treatment strategies. This is even more important since healthcare systems are increasingly challenged by expanding costs, shortage of medical professionals, and patients with increasing comorbidities [11]. So, the value of routinely performing SNRBs needs to be weighted not only in a clinical but also in a value based health‐economic perspective [22].

### 4.4 Recommendations for Future Clinical Practice and Research

This systematic review underlines the absence of RCTs to demonstrate clinical usefulness of prognostic SNRBs in the preparation of patients towards PRF treatment for chronic radicular pain. The lack of robust evidence in combination with increasing challenges in future healthcare urges policy makers and scientific communities to induce large value based randomized prospective studies to explore and evaluate the best possible clinical pathways and algorithms for patients with chronic radicular pain. We recommend future multi‐center RCTs in patients with chronic lumbosacral, thoracic and cervical radicular pain, comparing PRF alone to PRF preceded by a prognostic SNRB with local anesthetics. Besides the role of prognostic SNRBs, alternative prognostic strategies may need to be investigated, such as selective radiofrequency stimulation of the dorsal root ganglion [23, 24].

In addition, for future research, an urgent appeal has to be made to standardize and uniformize nomenclature around diagnosis, interventional pain management and outcome evaluation in these patients. In this systematic review, multiple variations of SNRBs (diagnostic, prognostic and therapeutic; different volumes and types of injectate) came to light which undoubtedly impede research and patient care on this topic. In case of SNRBs, the authors suggest to strictly standardize the technical performance of the intervention according to international guidelines and specify the nature of the SNRB by using additional terms as diagnostic, prognostic and therapeutic.

## Author Contributions

All authors have made significant scientific contributions to the work, are familiar with the content, and have approved the final version of this manuscript.

## Funding

The authors have nothing to report.

## Ethics Statement

The authors have nothing to report.

## Consent

The authors have nothing to report.

## Conflicts of Interest

Kris Vissers and Koen van Boxem are editorial board members of Pain Practice and co‐authors of this article. The other authors do not have any conflicts of interest.

## Supporting information


**Appendix S1:** papr70132‐sup‐0001‐DataS1.docx.

## Data Availability

Data sharing not applicable to this article as no datasets were generated or analyzed during the current study.
