# Supplementary material for: Prognostic Value of Selective Nerve Root Blocks Prior to Pulsed Radiofrequency in the Treatment of Patients With Chronic Radicular Pain: A Systematic Review
Source: Pain Pract. 2026 Mar 6;26(3):e70132. doi: 10.1111/papr.70132 (PMC12963952; doi:10.1111/papr.70132)
Supplement: Supplementary file 1 — Appendix S1: papr70132‐sup‐0001‐DataS1.docx. [file PAPR-26-0-s001.docx]

**APPENDIX A**

Search

1. PubMed

("Spinal Nerve Roots"[Mesh] OR nerve root*[tiab] OR dorsal root gangli*[tiab] OR spinal gangli*[tiab] OR spinal root*[tiab])

AND

("Pulsed Radiofrequency Treatment"[Mesh] OR radiofreq*[tiab] OR PRF[tiab])

AND

("Predictive Value of Tests"[Mesh] OR predict*[tiab] OR prognostic*[tiab] OR diagnostic*[tiab] OR selective[tiab] OR neurotom*[tiab])

AND

("Radiculopathy"[Mesh] OR radiculopath*[tiab] OR radiculo path*[tiab] OR radicular pain*[tiab] OR radicular syndrome*[tiab] OR low back pain[tiab] OR "Low Back Pain"[Mesh] OR sciatic*[tiab])

1. Embase

(Spinal Nerve/ or dorsal root/ or sciatic nerve/ or spinal ganglion/ or spinal root/ or nerve root/ OR nerve root*.ti,ab,kf. OR dorsal root gangli*.ti,ab,kf. OR spinal gangli*.ti,ab,kf. OR spinal root*.ti,ab,kf. or sciatic nerve*.ti,ab,kf. )

AND

(Pulsed Radiofrequency Treatment/ OR radiofreq*.ti,ab,kf. OR PRF.ti,ab,kf.)

AND

(prediction/ or predictive value/ or prognosis/ or prognostic*.ti,ab,kf. OR diagnostic*.ti,ab,kf. OR selective.ti,ab,kf. OR neurotom*.ti,ab,kf.)

AND

(radiculopathy/ or "nerve root lesion"/ or polyradiculoneuropathy/ or radicular pain/ OR radiculopath*.ti,ab,kf. OR radiculo path*.ti,ab,kf. OR radicular pain*.ti,ab,kf. OR radicular syndrome*.ti,ab,kf. OR low back pain.ti,ab,kf. OR Low Back Pain/ OR sciatic*.ti,ab,kf.)

1. Cochrane

([mh "Spinal Nerve Roots"] OR ("nerve" NEXT root*):ti,ab,kw OR ("dorsal root" NEXT gangli*):ti,ab,kw OR ("spinal" NEXT gangli*):ti,ab,kw OR ("spinal" NEXT root*):ti,ab,kw)

AND

([mh "Pulsed Radiofrequency Treatment"] OR radiofreq*:ti,ab,kw OR PRF:ti,ab,kw)

AND

([mh "Predictive Value of Tests"] OR predict*:ti,ab,kw OR prognostic*:ti,ab,kw OR diagnostic*:ti,ab,kw OR selective:ti,ab,kw OR neurotom*:ti,ab,kw)

AND

([mh Radiculopathy] OR radiculopath*:ti,ab,kw OR ("radiculo" NEXT path*):ti,ab,kw OR ("radicular" NEXT pain*):ti,ab,kw OR ("radicular" NEXT syndrome*):ti,ab,kw OR "low back pain":ti,ab,kw OR [mh "Low Back Pain"] OR sciatic*:ti,ab,kw)
